# Monitoring the Status and Trends of Tropical Forest Terrestrial Vertebrate Communities from Camera Trap Data: A Tool for Conservation

**DOI:** 10.1371/journal.pone.0073707

**Published:** 2013-09-04

**Authors:** Jorge A. Ahumada, Johanna Hurtado, Diego Lizcano

**Affiliations:** 1 Tropical Ecology Assessment and Monitoring Network, Betty and Gordon Moore Center for Science and Oceans, Conservation International, Arlington, Virginia, United States of America; 2 La Selva Biological Station, Organization for Tropical Studies, Sarapiqui, Costa Rica; 3 Grupo de Investigación en Ecología y Biogeografía (GIEB), Universidad de Pamplona, Pamplona, Colombia; University of Pretoria, South Africa

## Abstract

Reducing the loss of biodiversity is key to ensure the future well being of the planet. Indicators to measure the state of biodiversity should come from primary data that are collected using consistent field methods across several sites, longitudinal, and derived using sound statistical methods that correct for observation/detection bias. In this paper we analyze camera trap data collected between 2008 and 2012 at a site in Costa Rica (Volcan Barva transect) as part of an ongoing tropical forest global monitoring network (Tropical Ecology Assessment and Monitoring Network). We estimated occupancy dynamics for 13 species of mammals, using a hierarchical modeling approach. We calculated detection-corrected species richness and the Wildlife Picture Index, a promising new indicator derived from camera trap data that measures changes in biodiversity from the occupancy estimates of individual species. Our results show that 3 out of 13 species showed significant declines in occupancy over 5 years (lowland paca, Central American agouti, nine-banded armadillo). We hypothesize that hunting, competition and/or increased predation for paca and agouti might explain these patterns. Species richness and the Wildlife Picture Index are relatively stable at the site, but small herbivores that are hunted showed a decline in diversity of about 25%. We demonstrate the usefulness of longitudinal camera trap deployments coupled with modern statistical methods and advocate for the use of this approach in monitoring and developing global and national indicators for biodiversity change.

## Introduction

Reducing the loss of biodiversity is key to ensuring the future well-being of our planet and humanity [1]. The parties to the Convention on Biological Diversity (CBD) proposed a plan to reduce the rate of biodiversity loss by 2020 (Decision X/2: Strategic Plan for Biodiversity 2011–2020). This plan outlines 20 targets to evaluate progress (the Aichi Biodiversity Targets). Measuring progress towards these targets requires data and synthetic indicators (see [2] for an indicator analysis up to 2010). For example, to prevent the extinction of threatened species (Target 12) and ensure adequate protection of terrestrial, fresh water and marine areas (Target 11), countries need indicators that measure trends in abundance and distribution of species; protected area management effectiveness; and extinction risk of species. Currently, these and other indicators are assembled from a variety of available data collected for different objectives and questions, using different methodologies, at different spatial and temporal scales, and often not derived from longitudinal studies and with inadequate metadata. These constraints encumber the construction, interpretation and robustness of indicators, and therefore their usefulness, in evaluating progress towards the Aichi Biodiversity Targets [3]. Answering the relatively simple question of whether a species is increasing, decreasing or stable in time at a site or in a country, can be hindered by the quality and consistency of the data that inform the indicators.

Ideally, species information for monitoring indicators should come from primary data collected using consistent methodologies that can be deployed at a wide range of spatial and temporal scales, and made available in near-real time. Indicators using primary data that meet these criteria would be less biased and more precise than indicators derived from secondary and summary data [4]. For example, the status of many forest terrestrial vertebrate species is assessed using various methodologies ranging from expert opinion to systematic field assessments such as line transects, point counts and capture/recapture studies. Some of these field methodologies are well developed but hard to replicate and standardize due to inadequate and inconsistent training, observation bias, differences in sampling effort, and other sampling factors.

Camera traps are a useful, efficient, cost/effective, easily replicable tool to study and monitor ground-dwelling terrestrial mammals and birds [5] [3], [6]–[9]. In comparison with other field sampling methods, they are well suited to standardization, since human influence and error are reduced to placement and maintenance of the traps and identification of the photographs. With the arrival of digital camera traps in the last decade, and their increased affordability, many projects have started using them as tools for assessing and inventorying terrestrial vertebrates, especially in forests, where visibility is reduced and encounter rates with medium and large terrestrial vertebrates are often low. If camera trap deployments are designed correctly, they yield extremely valuable information about the terrestrial vertebrate community, including species diversity, species occupancy and abundance, and community structure [5], as well as species activity budgets, behavior and movements [10]. They are also a valuable tool for monitoring since camera trap deployments can be replicated seasonally or annually under the same field sampling conditions [11]. Additionally, camera trap data offers the opportunity to separately model the ecological state variable of interest (e.g., abundance or probability of occurrence of a species) while taking into account the detection process (e.g., the probability of detecting a species given that it occurs at the site) [12]–[14]. This allows for unbiased indicator estimation, making camera trap surveys extremely useful for monitoring programs aimed at measuring progress towards biodiversity conservation targets (such as Aichi Target 12).

In this paper, we use camera trap data that is regularly collected by the Tropical Ecology Assessment and Monitoring (TEAM) Network along the Volcan Barva Transect, Costa Rica, to demonstrate how these data can be used to calculate temporal species indicators for various mammal species of interest in the area and the larger community. We uncover declines in some species, propose mechanisms to explain these, and suggest management actions to reverse these patterns. We also use the camera trap information to estimate detection-corrected indicators of species richness for the community, as well as the Wildlife Picture Index (WPI), an increasingly useful indicator for assessing the status of communities of vertebrates monitored with camera trap data [15]. We propose a novel approach to calculate the WPI and show how it can be disaggregated for different groups of species (hunted vs. not hunted; different functional groups) to examine diversity trends for these groups and more effectively manage them.

## Methods

### Study site

**Figure 1:**
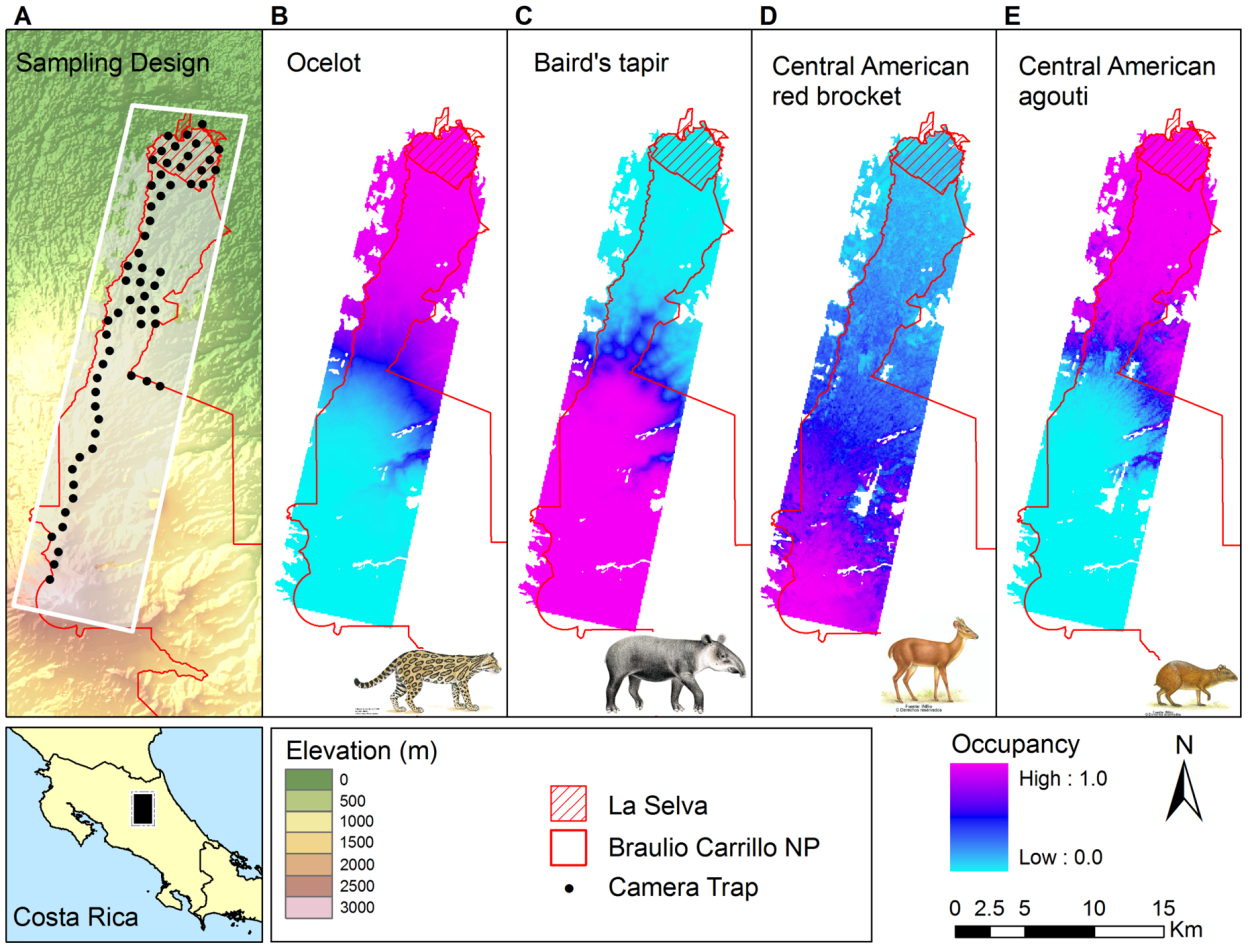
Maps showing the sampling design and modeled occupancy for species where occupancy depended on covariates. A. Distribution of camera trap sampling points along the Volcan Barva transect, Costa Rica. B,C,D,E: Modeled occupancy for Ocelot, Baird's tapir, Central American red brocket and Central American agouti at the baseline year (2008).

We collected data along a 30 km continuous strip of forest spanning a 3000 m altitudinal gradient between “La Selva” Biological Station, a small private ecological reserve (16 km^2^), and the larger Braulio Carrillo National Park (460 km^2^) in north-eastern Costa Rica (Figure 1A). This area, referred to as the Volcan Barva TEAM site, contains 280 km^2^ of lowland tropical forest, montane tropical forest, and cloud forest surrounded by a matrix of plantations and pastures.

The study did not involve any collection of animal specimens in the field; only photographic images. To work in Braulio Carillo National Park we obtained a “scientific passport” permit (# 04511) issued by the Costa Rican National System of Conservation Areas (Sistema Nacional de Areas de Conservación–SINAC) in 2003. To work at La Selva Biological Station we received a research permit approved by La Selva Academic Committee (also #04511) in December 2003. Both permits are still in effect as of May 1, 2013.

### Camera trap data collection

We deployed camera traps in the field at the Volcan Barva TEAM site from 2008 until 2012 using the standardized TEAM Terrestrial Vertebrate Monitoring Protocol [11]. We deployed 60 camera trap sampling points (one camera per point) along the altitudinal gradient at Volcan Barva TEAM site at a density of 1 camera/km^2^, away from main trails and with no bait. Each year, camera traps were deployed in three sequential 20-point sampling arrays, each camera active for at least 30 days during the drier months of the year (January to May). The same sampling points were used every year (and continue to be monitored). At the end of each deployment, memory cards were recovered, and images were processed and identified using specialized software – DeskTEAM [16]. Animals were identified following International Union for the Conservation of Nature (IUCN) taxonomic authority sources for mammals [17] and following the mammal taxonomy of the IUCN Red List. Further details are available on the equipment used and camera trap settings for the TEAM Terrestrial Vertebrate Monitoring Protocol [11]. All data were uploaded to the TEAM portal and are publicly available at http://www.teamnetwork.org. A total of 22437 images were recorded between 2008 and 2012, resulting from an effort of 8725 camera trap sampling days (see Table S1 in File S1 for details).

#### Selection of species

Out of 26 mammal species detected at Volcan Barva between 2008 and 2012, we focused our analysis on 13 target species: collared peccary, cougar, jaguarundi, jaguar, margay, ocelot, Central American agouti, coati, lowland paca, Baird's tapir, tayra, Central American red brocket and nine-banded armadillo. These species were selected based on three non-mutually exclusive criteria: 1) species commonly detected in the study area; 2) species with conservation interest or some level of threat or vulnerability according to the IUCN Red list or known to be hunted locally; and 3) species with key ecological functions in the ecosystem as prey, seed dispersers or predators. The full list of 26 species can be found in the supporting information (Table S2 in File S1 ).

#### Data preparation

The raw observation records (a camera trap image detection with its associated metadata) were downloaded from the TEAM portal (data package id: TV-20130322130624_4502). These data were condensed into presence/absence matrices, one for each species and each year, where the rows correspond to sampling points and the columns are time periods (days). The cells in these matrices are either 1 (the species was photographed at the given sampling point and day), 0 (the species was not photographed) or NA (the point was not actively sampled during this day). To reduce model computation time and increase efficiency, we grouped observations into 15 time periods for each species and each year (each time period equivalent to about 7–8 days of sampling). Observations remained as 0's and 1's after grouping. This does not affect the model estimates and only changes the units of the estimated detection probabilities for each species (see below). All data processing was done using scripts programmed in the language R [18].

#### Modeling approach

We used occupancy (estimated probability of occurrence of the species at the site) as our metric of abundance for each species. From the binary matrices, we estimated the occupancy of each species each year, by fitting dynamic occupancy models [12]. These models are hierarchical; the ecological process that influences occupancy is modeled separately from the observation process. In the ecological process model the presence/absence of a species *i* at a camera trap point *j* in the first year of observation, is an unobserved latent variable *Z_ij1_* resulting from a Bernoulli process with expected probability ψ*_ij1_*: *Z_ij1_* ∼ Bernoulli(ψ*_ij1_*). For the first year of observations the occupancy itself (ψ*_ij1_*), can be modeled as a function of sampling point covariates using a logistic link − logit(ψ*_ij1_*)  =  βX*_ij_* − where X*_ij_* is the design matrix of covariates, and β is a vector of parameters to be estimated. For subsequent years (year >1), the site remains occupied by the species with probability *φ,* or goes locally extinct from that site with probability (1-*φ*), where *φ* is the apparent survival of the species from one year to the next and (1-*φ*) is the local extinction probability. If the species did not occupy site *j* in year *t* (*Z_ijt_*  = 0), it can colonize this site by the following year *t +1* with probability *γ*. The dynamics of a species *i* can be described recursively for any two contiguous years as:
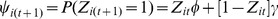



The survival probability and the colonization probability can also be modeled as a function of covariates (sampling point covariates or yearly covariates) using a logit link as described above.

The observation process of the model assumes the observations for each species *i* at each sampling point *j*, year *t*, and observation period *k*, *y_ijtk_*, as realizations of a Bernoulli process with mean *Z_it_ p_itk_* where *p_itk_* is the detection probability of species *i* at year *t*: *y_ijtk_ ∼* Bernoulli(*Z_it_ p_itk_*). This takes into account imperfect detection at the sampling point (false negatives) and provides an unbiased estimate of occupancy. As with the other parameters, *p* can be modeled as a function of sampling point covariates, yearly covariates, and additionally, within-year temporal observation covariates (e.g., temperature or day length at each of the 15 observation periods within a year). Under this model it is assumed that there is closure within years, but not between years (colonization and extinction only happen between years).

#### Model covariates

For this analysis we only used sampling point covariates and yearly covariates. The following covariates were calculated at each camera trap point: canopy height, aspect, slope, elevation, forest type and distance to edge. Canopy height, elevation, aspect and slope were derived from NASA's Land Vegetation and Ice Sensor (LVIS) laser altimetry [19] collected in year 2005. The LVIS point cloud data was interpolated using ArcGIS 10.1 (Environmental Systems Research Institute 2012) natural neighbor algorithm. From this interpolation we ws was obtained a 1-m resolution digital surface model (DSM) and a digital height model (DHM) with the same spatial resolution (1 m). Aspect and degree of slope were derived using the standard ArcGIS algorithms from the DSM. Forest type was assessed by identifying representative training areas of young forest through a comparison between two multispectral images (Landsat TM and Landsat ETM+) from February 1986 and January 2012. Areas of young forest were selected using ENVI software (Exelis Visual Information Solutions, Boulder, Colorado) to detect change and to visualize those areas with more change. For identifying old forest training sites, we use nine well-known 1 ha vegetation plots of primary forest located across the elevational gradient at La Selva Biological Station. These plots were stratified in the standard Holdridge life zones [20], and then grouped into three categories: low, middle and high elevation. We also included a special forest class –slope– in areas where the slope was higher than 60 percent. We then performed a supervised classification of forest types using training sites and independent and validated data to generate a map of six classes of forest types according to forest age and elevation: Slope, Primary-low elevation, Primary-intermediate elevation, Primary-high elevation, Primary-intermediate/high elevation, and Secondary. To obtain the distance from the camera trap point to the nearest forest edge, we overlapped the points with a map of 10 m buffer classes from the forest polygon boundary. This distance map was then generated in ArcGIS using the function buffer.

#### Model fitting

To find the best multi-seasonal occupancy model for each species, we first did an exploratory analysis for several species with enough data (>15 site detections per year), by fitting logistic regression models on the observed occupancy (naïve, since it is not corrected for detection probability) with various degrees of complexity adding covariates on occupancy, extinction, survival and detection probability using a step-wise regression approach. These initial analyses revealed that colonization, survival and detection probability were fairly insensitive to covariates resulting in models with little support. We then fitted and tested a small set of candidate models using the hierarchical approach described above for each species. These included: 1) a ‘null-type’ model with no covariates on occupancy and with or without year-specific colonization, survival and detection probabilities, and 2) year-1 occupancy covariates with additive terms and no interaction terms. For species with low numbers of observations (<15 site detections/year) we fitted null-type models, while for other species we fitted both types of models. In addition to the individual species model, we also fitted a model where we grouped all cat species together (jaguar, cougar, ocelot, margay and jaguarundi) in an attempt to overcome the low numbers of detections obtained for each of these species individually [21]. We selected the best model using AIC model selection criteria [22]. Models were fitted using package **unmarked** in R [23]. We then refitted the most supported models using a Bayesian approach [24] using software JAGS [25] running through package **R2jags** in R [26]. This approach yields similar results to the likelihood-based approach used in **unmarked**, but works better with species with few observations (low detection probability, low occupancy or both) and adds some other benefits (see Community Dynamics below). JAGS models were based on existing code [24] and were run with 5 chains of 30,000 iterations each, a thinning rate of 3 (every third iteration discarded) and a burn-in rate of 20,000 iterations (ignored for calculation of posterior density distributions). Models were checked for convergence by visually inspecting parameter time series and by ensuring the Gelman-Rubin statistic for each parameter was close to 1 [27]. Model parameters where recovered using the median or the mode of the distribution as many of the posterior distributions were highly skewed. Highest posterior density intervals (HPDI) were extracted from the posterior distributions at a 95% level for inference purposes. Model fit was assessed by calculating two chi-square statistics and computing a Bayesian posterior predictive check (BPPC) on these statistics (M. Kéry, pers. comm.). The first chi-square (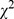
[obs]) calculates the discrepancy between annual, aggregated, observed detection frequencies vs. model detection frequencies at each model iteration. The second chi-square (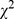
[new]) was calculated by generating a new realization using model parameters (‘perfect’ observation) at each model iteration, and then aggregating the data to annual detection frequencies and computing the discrepancy between this new prediction and the original model prediction. The BPPC is then calculated as the expected value of 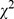
[new]> 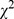
[obs] [27] (Bayesian *p* -value in Table 1). Values very close to 0 or 1 indicate lack of model fit. JAGS code for all the models (including calculation of BPPC) is available in the supporting information (Text S1, Text S2, Text S3, Text S4 and Text S5 in File S1 ).

**Table 1:** Species names, functional guilds, conservation status and summary model results for each species.

Binomial Name	Common Name	Functional group	IUCN Red List Status^1^	Best Model^2^	Model Dynamics	Bayesian p-value
*Cuniculus paca*	Lowland paca	small herbivore	LC	ψ(.)γ(year)φ(year)p(.)	Decreasing	0.364
*Dasyprocta punctata*	Central American agouti	small herbivore	LC	ψ(Ele+Can)γ(year)φ(year)p(.)	Decreasing	0.301
*Dasypus* *novemcinctus*	Nine-banded armadillo	omnivore	LC	ψ(.)γ(year)φ(year)p(year)	Decreasing	0.154
*Eira barbara*	Tayra	omnivore	LC	ψ(.)γ(.)φ(.)p(.)	Stable	0.215
*Leopardus pardalis*	Ocelot	carnivore	LC	ψ(Ele)γ(year)φ(year)p(.)	Stable	0.451
*Leopardus wiedii*	Margay	carnivore	NT	ψ(.)γ(year)φ(year)p(year)	Stable	0.531
*Mazama temama*	Central American red brocket	large herbivore	DD	ψ(Ele+Can)γ(year) φ(year)p(.)	Stable	0.255
*Nasua Narica*	White-nosed coati	omnivore	LC	ψ(.)γ(year)φ(year)p(.)	Stable	0.360
*Panthera Onca*	Jaguar	carnivore	NT	ψ(.)γ(year)φ(year)p(year)	Stable	0.531
*Pecari tajacu*	Collared peccary	large herbivore	LC	ψ(.)γ(year)φ(year)p(.)	Stable	0.054
*Puma concolor*	Cougar	carnivore	LC	ψ(.)γ(year)φ(year)p(year)	Stable	0.516
*Puma yaguaroundi*	Jaguarundi	carnivore	LC	ψ(.)γ(year)φ(year)p(year)	Stable	0.511
*Tapirus bardii*	Baird's tapir	large herbivore	EN	ψ(Ele+Edg)γ(year) φ(year)p(.)	Stable	0.484

1 LC  =  least concern; NT  =  near threatened; EN  =  endangered; DD  =  data deficient.

2 Model with the lowest Deviance Information Criterion (DIC). Model parameters: ψ  =  occupancy year 1; γ  =  apparent survival; φ =  colonization probability; p  =  detection probability. Model Covariates: Ele  =  elevation (m); Can  =  canopy height (m); Edg  =  distance to edge (m); year  =  year of measurement. A dot (.) means no covariates were added to this parameter.

Changes in occupancy from year *t* to year *t* + *n* (n = 1 to 4) were estimated by calculating the distribution of the ratio ψ(*t* + *n*)/ψ (*t*)  =  *λ_n_* as defined by MacKenzie et al. [12]. This resulted in a matrix of *λ* values describing the change between a given year *t* and any future year *t + n*. We considered that a decline in occupancy had occurred between year *t* and year *t + n* if the upper boundary of the HPDI of *λ_n_* <1. We considered an increase in occupancy during the same time period if the lower boundary of the HPDI of *λ_n_* >1. Otherwise (if 1 was included within the HPDI of *λ_n_*), we considered that no change had occurred between the two years.

#### Community dynamics

We calculated two metrics for community dynamics: species richness and the WPI [3]. Changes in species richness through time were calculated from the camera trap data using an occupancy approach [28], [29]. Occupancy (probability of a set of species being present in a given year, corrected by detection probability) is an estimate of relative species richness ψ_S_, or the proportion of species present at a site from a known regional pool of species. A regional pool of 40 species have been detected historically at Volcan Barva, excluding arboreal and flying mammals [30]. We constructed a species-by-sampling-point detection matrix for each year and used the same analysis rationale described above for dynamic models to estimate changes in relative species richness through time (species that were not detected at the site in a given year are coded in the matrix as zeros). We fitted two covariates to relative species richness: body size and trophic group. Body size was assigned from a global database of body sizes for mammals [31]. Trophic group was either herbivore, carnivore, omnivore or insectivore and was assigned based on typical dietary and ecological habits of each species. Models were fitted using package **unmarked** [32], and 95% confidence intervals for species richness were obtained by running a non-parametric bootstrap of the data with 500 iterations. Final values for species richness were obtained by multiplying relative species richness by the number of species in the regional pool (40).

We also calculated the Wildlife Picture Index (WPI), defined as the geometric mean of the occupancies of the 13 focal species scaled by their occupancies in the first year of the survey [3]. Unlike species richness, the WPI is an ideal metric for evaluating changes in biodiversity, because it is sensitive to changes in richness, relative abundance (occupancy), dominance and other measures of community diversity [33]. O'Brien et al.[3] calculated the WPI by bootstrapping species to estimate uncertainty in the occupancies and then fit Generalized Additive Models (GAMs) to the estimated occupancies [33]. We used a different and more direct approach since our Bayesian model fits gave us the posterior distribution of occupancy for each species each year. This allowed us to compute the WPI as a derived quantity from these distributions (geometric mean of the relative occupancies at each model iteration after model burn-in) resulting in a full posterior distribution for the WPI distribution each year. From these distributions we extracted the 95% HPDI and used the median or mode (whichever was lowest) as the measure of central tendency of the distribution. We contend this is a much more “natural” way of computing the WPI, since it comes directly from the underlying modeled occupancy distributions, which in turn come from fitting a dynamic model of occupancy [34].

Changes in WPI from year *t* to year *t* + *n* were estimated by calculating the distribution of the ratio WPI(*t* + *n*)/WPI(*t*) in a similar way defined for testing yearly differences between occupancies (see above).

In addition to the community-wide WPI, we also calculated the WPI for species grouped according to conservation status and functional groups. Following the IUCN Red List, we separated species of least concern from endangered, near threatened and data deficient species and recalculated the WPI using the approach above for each group. We also separated species into ‘hunted’ or ‘not hunted’ based on local information provided by park managers and patrol officers at the site. In addition, we separated species into four functional groups –carnivores, large herbivores (>20 kg), small herbivores (<20 kg), omnivores– and calculated the WPI for each. We calculated *λ_n_* for each of these as described above.

## Results and Discussion

### Species dynamics (spatial)

Table 1 summarizes the model fitting results for all 13 focal species examined (parameter values and standard errors for all parameters can be found in Table S3 in File S1 ). Six different model types adequately described the dynamics of these species, three of which had covariates on occupancy (elevation, elevation+canopy, elevation+edge). All models showed adequate fit as evidenced by the absence of Bayesian p-values too close to 0 or 1 (Table 1). Only four species were adequately fit by models with covariates in occupancy: one species responded to elevation alone (ocelot–Figure 1B), one species responded to elevation and edge (Baird's tapir–Figure 1C), and two species responded to elevation and canopy height (Central American agouti, Central American red brocket–Figures 1D,1E). Ocelot and Central American agouti occupancies decreased with elevation (agouti: β(elevation)  = −5.33, sd  = 1.8; ocelot: β(elevation)  = −4.56, sd  = 1.9), while Baird's tapir and Central American red brocket occupancies increased with elevation (tapir: β(elevation)  = 5.72, sd  = 1.7, brocket: β(elevation)  = 1.61, sd  = 1.0). Central American red brocket and Central American agouti occupancy increased with canopy height, but the effect was much stronger for agouti (brocket: β(canopy)  = 0.66, sd  = 0.6; agouti: β(canopy)  = 1.12, sd  = 0.9). Distance from the edge had a positive effect on the occupancy of Baird's tapir (β(edge)  = 1.37, sd  = 0.86).

We do not have yet a full understanding of why the occupancy of these species depends on spatial covariates, while the occupancy of other species with high number of detections does not (e.g., collared peccary, lowland paca). Two of the species with spatial-dependent occupancy are large herbivores (C. American red brocket, Baird's tapir) that are more common at middle and high elevations along Volcan Barva. The tapir seems to prefer areas away from forest edges, which might be also areas close to water, a habitat preference reported for the species [35]. Red brockets prefer habitats with closed high forest and high fruit density [36], explaining some of the effects of canopy height. Agoutis are known to prefer tall lowland forests with high palm species diversity and high number of palm nuts, their preferred food source [37], thus the elevation and canopy height effects on occupancy. The spatial distribution of ocelot's occupancy might be reflecting the spatial distribution of their main prey at this site – agoutis, pacas and armadillos [38]. The distribution of these species might also be the result of the interaction between habitat preferences and levels of hunting.

### Species Dynamics (temporal)

All species, except for tayra, were described by models with year-specific survival and colonization probabilities. Models for five species (nine-banded armadillo, margay, jaguar, cougar and jaguarundi) also included terms for year-specific detection probabilities.

**Figure 2:**
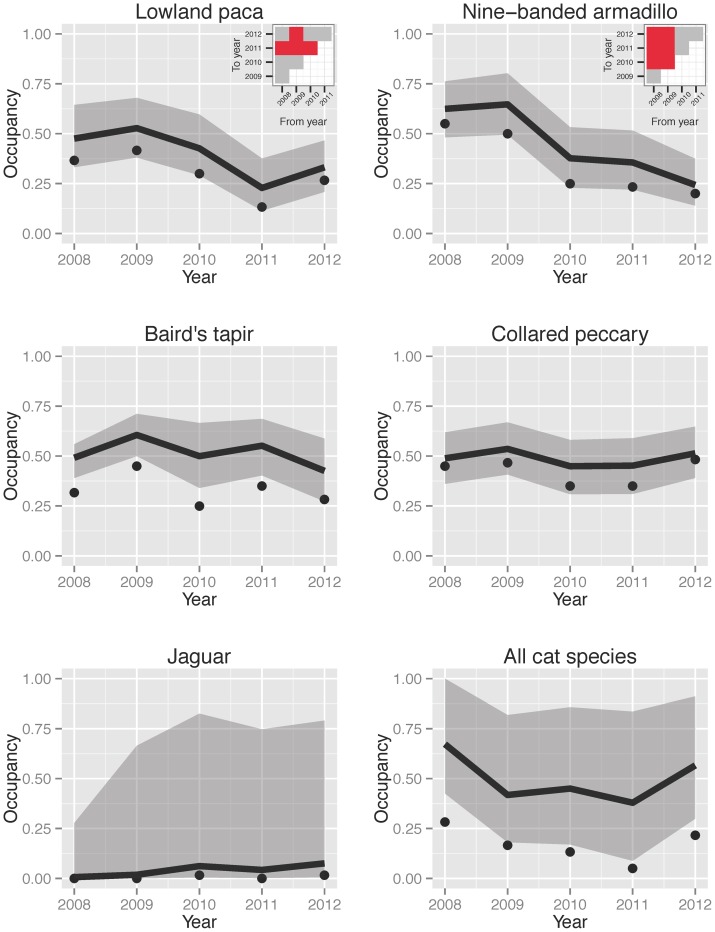
Temporal dynamics in occupancy for selected species. Occupancy dynamics for Lowland paca (A), Nine-banded armadillo (B), Baird's tapir (C), Collared peccary (D), Jaguar (E) and all cat species combined –Ocelot, Margay, Jaguar, Puma and Jaguarundi– (F). Observed occupancies (naive) are shown as points, and modeled occupancy is shown as a solid line (median or mode of the posterior distribution). The shaded gray region spans the highest posterior density intervals for occupancy. The small inset graphs in A and B symbolize the statistical signal of λ between any two particular years. Values of λ significantly smaller than 1 are symbolized in red; values not significantly different from 1 are symbolized by grey. These are not shown for panels C,D,E and F because λ was not significantly different from 1 between years for any of these species.

Three species showed significant declines in occupancy through time (Table 1): lowland paca, Central American agouti and nine-banded armadillo (henceforth paca, agouti and armadillo for simplicity). Figures 2A and 2B show the temporal dynamics of paca and armadillo with year-to-year assessments of change (λ) (agouti dynamics are similar to paca –not shown). Paca showed some stability in occupancy during the first 2 years of monitoring but then a significant decline through 2011 with an apparent recovery in 2012. Armadillo showed a more sharp decline relative to the initial year of monitoring, but again, some stability in the last two years (2011–2012).

Five remaining species with enough detection data (>15 detections/year) showed no temporal changes in occupancy through time (ocelot, Central American red brocket, collared peccary, Baird's tapir, white-nosed coati). Figure 2C and 2D show the yearly dynamics of Baird's tapir and collared peccary as representative species of this group.

The remaining five species with <15 detections per year (tayra, margay, jaguar, cougar and jaguarundi) showed no consistent changes in occupancy, but the modeled occupancies are not precise enough to detect change in a short 5-year time span. Figure 2E shows the temporal dynamics of a typical species in this group (jaguar) with very low, modeled occupancies and wide, posterior density intervals. However, the temporal dynamics of all cat species combined suggests that this group has remained relatively stable during the five years of monitoring (Figure 2F).

From a conservation point of view, it is encouraging that most of the species analyzed at Volcan Barva showed apparent stability in occupancy during the first few years of monitoring. For the three species that show evidence of declines, we put forward several hypotheses to explain this pattern. Paca and agouti, but not armadillo, are coveted targets for hunters living around La Selva and Braulio Carillo National Park (O. Vargas, pers. comm.). We hypothesize that the patterns observed in paca and agouti can be the result of three different –and not necessarily mutually exclusive– mechanisms: 1) increased hunting pressure on these species; 2) competition for seeds between these species and other ubiquitous seed predators; and 3) increases in predator densities in the area.

Anecdotal hunting observations from park rangers at La Selva suggest that declines in these two species may be the result of increased hunting pressure at La Selva and Braulio Carrillo NP [39] (A. Ezeta, pers. comm.). Both species, as well as peccaries, are coveted by hunters, and park rangers often find traps, tracks and other evidence of hunting. However, hunting pressure might decrease when a species reaches some lower threshold, inducing a predator-prey-like cycle where the species recovers until its abundance is high enough to be easily hunted again. With the high rate of decline in occupancy in these species (50% decline on a span of 3–4 years), we predict that occupancy might continue to decline if hunting is the main driver and it is not controlled. We need 3–4 years of additional monitoring data to test this hypothesis.

Agoutis are seed predators that might be competing with collared peccaries for limited food resources. There is some evidence of seed competition between peccaries and agoutis at La Selva [40], with agoutis being less efficient at removing seeds than peccaries. Pacas feed on soft fruits rather than seeds, so they are unlikely to compete with agoutis, but likely competing with peccaries who are also fruit eaters. The remaining question is why is this pattern operating now? This might be related to increases in peccary populations in the last 10–15 years, but unfortunately no data are available for this region.

Another process that might be at work behind the declines of these two species is an increase in predators (wild cats) within the protected area resulting from deforestation and fragmentation after the expansion of Braulio Carrillo NP in 1986 [41]. However, our data suggests that cat species occurrence has not increased within the protected area over the last five years (Figure 2F). Nevertheless, data is sparse with wide confidence intervals, limiting what we can infer.

Currently, we are unable to explain the declining occupancies of armadillos at Volcan Barva (Figure 2B). Armadillos are not coveted by hunters in this area (O. Vargas, A. Ezeta pers. comm.), nor is there evidence for predator increases in the area (Figure 2F). Armadillos are habitat generalists found not only in forests, but also in open areas such as savannas and agricultural and rural areas [42] [43] (J. Hurtado, pers. comm.). Perhaps recent disturbances around the protected area, in conjunction with maturation of the forest in La Selva, have induced armadillos to move outside of the old-growth forest and into a more disturbed habitat matrix. However, visual comparison of LANDSAT images covering the Volcan Barva transect in 2008 and 2012 suggests no major changes in the forest matrix around the park (M. Rosa, pers.comm.).

### Community dynamics

**Figure 3:**
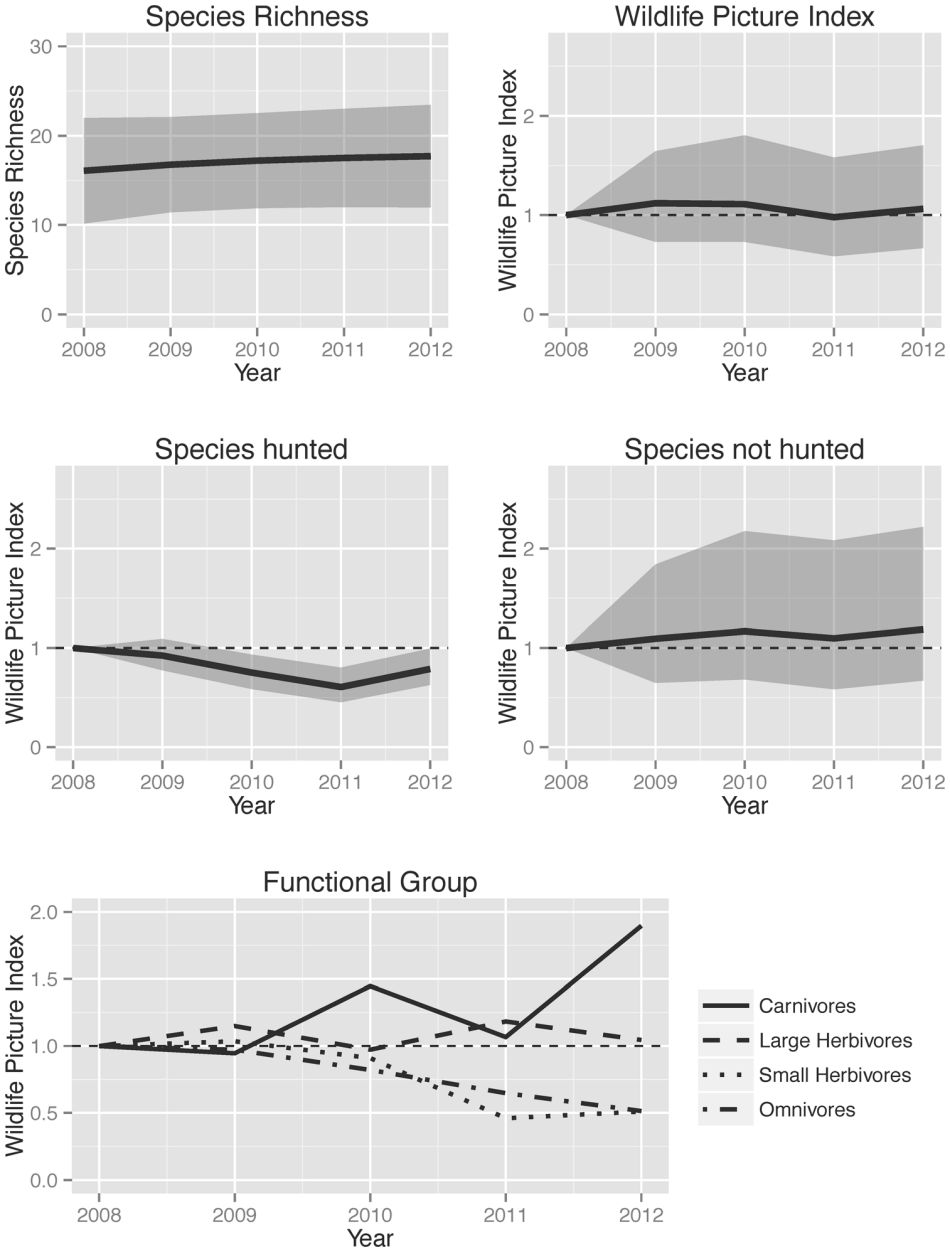
Comparison of different metrics of community dynamics. A. Modeled changes in mammal species richness at Volcan Barva transect, Costa Rica. The solid line shows the estimated value of richness and the shaded grey region spans the 95% confidence limits of the estimated distribution. B. Temporal changes in the Wildlife Picture Index (WPI) of 13 mammal species at Volcan Barva. The solid line is the mode of the posterior distribution of the WPI for each year and the shaded area spans the highest posterior density intervals for the WPI. Temporal changes in the WPI when it is disaggregated for species that are coveted by hunters (C) –Lowland paca, Central American agouti, Collared peccari– and species that are not normally hunted in the area (D) –all other species. E. Temporal changes in the WPI when it is disaggregated by functional groups of species; high posterior densities for each group are not shown for simplicity. The only group with a WPI significantly smaller than 1 in 2012 is the small herbivore group.

The yearly dynamics of species richness was best described by a model with covariates on species detection probability. Body size, functional guild, and year were significant covariates of detection, resulting in a model with the lowest AIC value (see supporting information –Table S4 in File S1 – for full model parameters and standard errors). Detection probability covaried positively with body size and differed between guilds (herbivores the highest and omnivores the lowest), with remaining variation in detection probability explained by the year term (detection estimate was about the same every year). Estimated species richness did not show significant trends between 2008–2012 (Figure 3A). Richness varied between 16 and 18 species +/−5 species (standard error).

The WPI combining all 13 species for the same period also showed only small deviations from the baseline and no significant changes in diversity between years (Figure 3B). However, the WPI of species known to be hunted at Volcan Barva (paca, peccary, agouti) showed a significant decrease in diversity compared to the WPI for species that are not hunted (Figure 3C, 3D). This pattern is mostly driven by declines in agoutis and pacas, since peccaries showed no significant trend (Figure 2D).

The declines in agoutis and pacas suggest that existing management practices to control hunting along the Volcan Barva transect might not be adequate to maintain these two species. However, as discussed above, agoutis and pacas may be competing with each other and with peccaries [40]. If competition with peccaries alone is driving these declines, we expect that occupancy of pacas and agoutis will remain low, while peccary occupancy will remain high or increase. If, on the other hand, hunting is the main mechanism driving these patterns, both species might show recoveries if hunting control becomes more effective, or, in the absence of hunting control, oscillations over the long run as hunting pressure decreases when they become rare and increases when the populations recover. Whichever mechanism is in place, minimizing hunting of these species is a good management practice since regular hunting increases mortality in addition to other mechanisms that might be keeping these populations at low levels.

We also calculated the WPI for different functional guilds of species, allowing us to examine dynamic changes in diversity separately for these groups. Although not relevant here, this analysis is particularly useful when comparing sites with different species assemblages, e.g., from different continents. Not surprisingly, the small herbivores (agoutis, pacas), were the only group to show significant declines in diversity, while the other groups showed no trend (Figure 3E). Separating the WPI for species with different conservation status, did not show any clear pattern. Some species of Least Concern (agoutis, pacas and armadillos) drove the WPI below 1, but other species in the same status pushed it back over 1, with no significant trend. The only endangered species in the community (Baird's tapir) showed no significant trend, an encouraging sign for this species.

We have demonstrated how camera trap data using a combination of consistent field methodology and appropriate statistical models that account and correct for possible detection bias and that explicitly incorporate ecological factors [12], [24] can yield useful and transparent indicators for species and community trends. Temporal changes in occupancy adequately capture the dynamics of species accounting for ecological processes as well as observation error and false negative detections. This approach allows for a better understanding and assessment of both the temporal (Figure 2) and spatial (Figure 1) dynamics of these species and is not limited to just occupancy; point abundance can also be used as a state metric by fitting N-mixture models [44]. Using these analytical methods, we can derive unbiased estimates of occupancy or point abundance for even the rare species, although the confidence around these estimates is broad. Out of the 13 species detected, only three showed significant declines in occupancy in our first assessment of this vertebrate monitoring program at Volcan Barva. Future monitoring data will allow us to further understand what mechanisms underlie these declines, but we are also proactively using these results to recommend conservation actions to park authorities at this site (placing a stronger emphasis on hunting control, in particular for pacas and agoutis).

We also estimated community diversity indices from camera trap data that take full advantage of the repeated observation process inherent in camera trapping. This results in species richness estimators that take into account detection probability and covariates that affect detection, occupancy, apparent survival and colonization [45], [46]. We also implemented the WPI, a more sensitive indicator of biodiversity that has many of the desired properties of biodiversity indices designed to detect change and measure progress towards local, regional and global biodiversity targets [3], [33]. Furthermore, the WPI can be disaggregated for different groups of species in the community or aggregated up to regional, continental and global scales. The WPI of this community is relatively stable, but the WPI for species that are hunted shows declines in biodiversity of 30–40% from the initial baseline explained by the decline in the two main small herbivores in the community (agoutis and pacas).

We contend that the WPI should be adopted as an indicator to measure progress towards some of the Aichi Biodiversity Targets (in particular Target 12, but also 4, 5, 7, 10, 11 and 15) ensuring that the underlying data and analytical methods for its calculation incorporate the best standards to overcome bias and increase precision. In 2013, the TEAM network, in conjunction with partners, will release the first global WPI assessment for tropical ground-dwelling mammals and birds derived from the largest global camera trap network in tropical forests (currently 16 sites), using the methods outlined here. In addition, at the level of a country, it is feasible and cost effective to set up similar camera trap networks to monitor key species across different habitats with the goal of producing a national-level WPI. Compared to more traditional survey methods for ground vertebrates (line transects) camera trap surveys are 15% and 30% cheaper to implement in forests and savannas respectively [15] with the added benefits of methodological standardization and ease of implementation. Current technological advances in camera trap technology, will continue to make camera traps cheaper in the future. The current statistical methods to derive the WPI and the underlying occupancies can all be implemented using open source software packages (**unmarked** for R, Presence, WinBUGS, JAGS) but semi-automated algorithms to estimate the WPI are under development to facilitate its calculation at larger temporal and spatial scales.

By monitoring the status and trends of different components of biodiversity –not just forest area– using standardized open methodologies and applying solid analytical techniques to these data, we will not only contribute to measuring progress towards our commitments to reduce biodiversity loss through the CBD Aichi Targets, but also provide actionable scientific information for on-time management decisions.

## Supporting Information

File S1 **This file contains Table** **S1-S4 and Text** **S1-S5.** Table S1, Details of camera trap deployments at Volcan Barva. Table S2, Full list of species captured in camera traps at Volcan Barva between 2008 and 2012. Table S3, Parameters from each model (sd of parameter) for all species. Table S4, Parameter and effects for species richness model. Text S1, JAGS/WinBUGS code for model ψ (.)γ(year) Φ (year)p(.) listed in Table 1. Text S2, JAGS/WinBUGS code for models ψ (Ele+Can)γ(year)Φ(year)p(.) and ψ (Ele+Edg)γ(year) Φ (year)p(.) listed in Table 1. Text S3, JAGS/WinBUGS code for model ψ (.)γ(year) Φ (year)p(year) listed in Table 1. Text S4. JAGS/WinBUGS code for model ψ(.)γ(.)Φ (.)p(.) listed in Table 1.
(DOCX)
