# Supplementary material for: Monitoring the Status and Trends of Tropical Forest Terrestrial Vertebrate Communities from Camera Trap Data: A Tool for Conservation
Source: PLoS One. 2013 Sep 4;8(9):e73707. doi: 10.1371/journal.pone.0073707 (PMC3762718; doi:10.1371/journal.pone.0073707)
Supplement: File S1 — This file contains Table S1-S4 and Text S1-S5. Table S1, Details of camera trap deployments at Volcan Barva. Table S2, Full list of species captured in camera traps at Volcan Barva between 2008 and 2012. Table S3, Parameters from each model (sd of parameter) for all species. Table S4, Parameter and effects for species richness model. Text S1, JAGS/WinBUGS code for model ψ (.)γ(year) Φ (year)p(.) listed in Table 1. Text S2, JAGS/WinBUGS code for models ψ (Ele+Can)γ(year)Φ(year)p(.) and ψ (Ele+Edg)γ(year) Φ (year)p(.) listed in Table 1. Text S3, JAGS/WinBUGS code for model ψ (.)γ(year) Φ (year)p(year) listed in Table 1. Text S4. JAGS/WinBUGS code for model ψ(.)γ(.)Φ (.)p(.) listed in Table 1. (DOCX) [file pone.0073707.s001.docx]

**Table S1. Details of camera trap deployments at Volcan Barva**. The number of camera traps column indicates the actual number of camera traps that had useful information at the end of the season; some camera traps were vandalized or experienced technical difficulties in the field. Sixty camera trap points were deployed every year.

| *Year* | *Start date* | *End date* | *# camera traps* | *Length of sampling (days)* | *Effort (camera trap – days)* |
| --- | --- | --- | --- | --- | --- |
| 2008 | 4/22/08 | 8/20/08 | 56 | 118 | 1878 |
| 2009 | 1/28/09 | 5/28/09 | 60 | 120 | 1913 |
| 2010 | 1/12/10 | 5/12/10 | 55 | 120 | 1554 |
| 2011 | 1/10/11 | 4/29/11 | 56 | 109 | 1543 |
| 2012 | 1/10/12 | 5/4/12 | 59 | 114 | 1837 |

**Table S2. Full list of species captured in camera traps at Volcan Barva between 2008 and 2012.**

| *Class* | *Family* | *Genus* | *Species* |
| --- | --- | --- | --- |
| Mammalia | Cervidae | *Mazama* | *temama* |
|  |  | *Odocoileus* | *virginianus* |
|  | Cuniculidae | *Cuniculus* | *paca* |
|  | Dasypodidae | *Dasypus* | *novemcinctus* |
|  | Dasyproctidae | *Dasyprocta* | *punctata* |
|  | Didelphidae | *Didelphis* | *marsupialis* |
|  |  | *Marmosa* | *mexicana* |
|  |  | *Metachirus* | *nudicaudatus* |
|  |  | *Philander* | *opossum* |
|  | Echimyidae | *Proechimys* | *semispinosus* |
|  | Erethizontidae | *Sphiggurus* | *mexicanus* |
|  | Felidae | *Leopardus* | *pardalis* |
|  |  | *Leopardus* | *wiedii* |
|  |  | *Panthera* | *onca* |
|  |  | *Puma* | *concolor* |
|  |  | *Puma* | *yagouaroundi* |
|  | Heteromyidae | *Heteromys* | *desmarestianus* |
|  | Mephitidae | *Conepatus* | *semistriatus* |
|  | Mustelidae | *Eira* | *barbara* |
|  | Myrmecophagidae | *Tamandua* | *mexicana* |
|  |  | *Tamandua* | *tetradactyla* |
|  | Procyonidae | *Nasua* | *narica* |
|  |  | *Procyon* | *lotor* |
|  | Sciuridae | *Sciurus* | *yucatanensis* |
|  |  | *Sciurus* | *variegatoides* |
|  |  | *Sciurus* | *granatensis* |
|  | Tapiridae | *Tapirus* | *bairdii* |
|  | Tayassuidae | *Pecari* | *tajacu* |
| Aves | Cracidae | *Crax* | *rubra* |
|  |  | *Penelope* | *purpurascens* |
|  | Momotidae | *Electron* | *platyrhynchum* |
|  | Odontophoridae | *Odontophorus* | *leucolaemus* |
|  | Tinamidae | *Tinamus* | *major* |

**Table S3. Parameters from each model (sd of parameter) for all species.**

|  |  | *Covariate coefficients (𝜓_1_ occupancy 1^st^ year)* | | | *Colonization probability (γ)* | | | |
| --- | --- | --- | --- | --- | --- | --- | --- | --- |
| Species | Model | β_0_ | β_1_ | β_2_ | γ_12_ | γ_23_ | γ_34_ | γ_45_ |
| *Cuniculus paca* | 𝜓(.)γ(year)ɸ(year)p(.) | – | – | – | 0.27 (0.115) | 0.25 (0.11) | 0.1 (0.073) | 0.19 (0.076) |
| *Dasyprocta punctata* | 𝜓(Ele+Can)γ(year)ɸ(year)p(.) | 0.47 (0.867) | -5.33 (1.796) | 1.12 (0.922) | 0.1 (0.074) | 0.07 (0.059) | 0.16 (0.077) | 0.32 (0.088) |
| *Dasypus novemcinctus* | 𝜓(.)γ(year)ɸ(year)p(year) | – | – | – | 0.42 (0.132) | 0.1 (0.077) | 0.14 (0.083) | 0.07 (0.052) |
| *Eira barbara* | 𝜓(.)γ(.)ɸ(.)p(.) | – | – | – | 0.07 (0.038) | = γ_12_ | = γ_12_ | = γ_12_ |
| *Leopardus pardalis* | 𝜓(Ele)γ(year)ɸ(year)p(.) | 0.89 (1.363) | -4.57 (1.877) | – | 0.25 (0.216) | 0.26 (0.228) | 0.22 (0.191) | 0.4 (0.203) |
| *Leopardus wiedii* | 𝜓(.)γ(year)ɸ(year)p(year) | – | – | – | 0.18 (0.272) | 0.21 (0.254) | 0.31 (0.272) | 0.44 (0.276) |
| *Mazama temama* | 𝜓(Ele+Can)γ(year)ɸ(year)p(.) | -1.17 (0.718) | 1.61 (1.001) | 0.66 (0.592) | 0.16 (0.072) | 0.16 (0.097) | 0.29 (0.124) | 0.14 (0.087) |
| *Nasua Narica* | 𝜓(.)γ(year)ɸ(year)p(.) | – | – | – | 0.16 (0.093) | 0.08 (0.077) | 0.04 (0.055) | 0.05 (0.045) |
| *Panthera Onca* | 𝜓(.)γ(year)ɸ(year)p(year) | – | – | – | 0.12 (0.217) | 0.25 (0.27) | 0.23 (0.268) | 0.31 (0.278) |
| *Pecari tajacu* | 𝜓(.)γ(year)ɸ(year)p(.) | – | – | – | 0.2 (0.079) | 0.18 (0.082) | 0.25 (0.089) | 0.24 (0.088) |
| *Puma concolor* | 𝜓(.)γ(year)ɸ(year)p(year) | – | – | – | 0.08 (0.078) | 0.32 (0.271) | 0.29 (0.269) | 0.3 (0.274) |
| *Puma yaguaroundi* | 𝜓(.)γ(year)ɸ(year)p(year) | – | – | – | 0.08 (0.089) | 0.3 (0.266) | 0.3 (0.274) | 0.32 (0.278) |
| *Tapirus bardii* | 𝜓(Ele+Edg)γ(year)ɸ(year)p(.) | 1.06 (0.967) | 5.73 (1.684) | 1.37 (0.861) | 0.27 (0.102) | 0.08 (0.071) | 0.2 (0.11) | 0.09 (0.078) |

|  | *Detection probability (p)* | | | | | *Survival probability (Φ)* | | | |
| --- | --- | --- | --- | --- | --- | --- | --- | --- | --- |
| Species | *p_1_* | *p_2_* | *p_3_* | *p_4_* | *p_5_* | *Φ_12_* | *Φ_23_* | *Φ_34_* | *Φ_45_* |
| *Cuniculus paca* | 0.27 (0.025) | = *p_1_* | = *p_1_* | = *p_1_* | = *p_1_* | 0.79 (0.108) | 0.62 (0.122) | 0.41 (0.134) | 0.81 (0.134) |
| *Dasyprocta punctata* | 0.35 (0.022) | = *p_1_* | = *p_1_* | = *p_1_* | = *p_1_* | 0.62 (0.089) | 0.67 (0.123) | 0.74 (0.123) | 0.71 (0.117) |
| *Dasypus novemcinctus* | 0.41 (0.041) | 0.27 (0.037) | 0.31 (0.061) | 0.24 (0.051) | 0.35 (0.064) | 0.78 (0.096) | 0.53 (0.116) | 0.72 (0.137) | 0.58 (0.135) |
| *Eira barbara* | 0.16 (0.065) | = *p_1_* | = *p_1_* | = *p_1_* | = *p_1_* | 0.33 (0.185) | = *Φ_12_* | = *Φ_12_* | = *Φ_12_* |
| *Leopardus pardalis* | 0.06 (0.014) | = *p_1_* | = *p_1_* | = *p_1_* | = *p_1_* | 0.66 (0.185) | 0.53 (0.23) | 0.26 (0.214) | 0.65 (0.237) |
| *Leopardus wiedii* | 0.16 (0.154) | 0.14 (0.227) | 0.12 (0.133) | 0.06 (0.083) | 0.05 (0.047) | 0.46 (0.299) | 0.48 (0.3) | 0.44 (0.287) | 0.45 (0.286) |
| *Mazama temama* | 0.26 (0.073) | 0.33 (0.065) | 0.18 (0.078) | 0.28 (0.052) | 0.24 (0.057) | 0.67 (0.15) | 0.64 (0.223) | 0.78 (0.147) | 0.63 (0.15) |
| *Nasua Narica* | 0.14 (0.039) | = *p_1_* | = *p_1_* | = *p_1_* | = *p_1_* | 0.53 (0.218) | 0.7 (0.197) | 0.43 (0.218) | 0.55 (0.244) |
| *Panthera Onca* | 0.38 (0.315) | 0.28 (0.307) | 0.09 (0.115) | 0.14 (0.24) | 0.06 (0.088) | 0.47 (0.293) | 0.46 (0.295) | 0.37 (0.292) | 0.43 (0.294) |
| *Pecari tajacu* | 0.45 (0.021) | = *p_1_* | = *p_1_* | = *p_1_* | = *p_1_* | 0.87 (0.073) | 0.68 (0.097) | 0.7 (0.116) | 0.85 (0.079) |
| *Puma concolor* | 0.13 (0.074) | 0.17 (0.078) | 0.06 (0.067) | 0.08 (0.124) | 0.05 (0.079) | 0.67 (0.22) | 0.48 (0.284) | 0.35 (0.283) | 0.42 (0.289) |
| *Puma yaguaroundi* | 0.13 (0.073) | 0.17 (0.077) | 0.06 (0.071) | 0.08 (0.123) | 0.05 (0.08) | 0.67 (0.216) | 0.46 (0.281) | 0.35 (0.285) | 0.41 (0.289) |
| *Tapirus bardii* | 0.21 (0.019) | = *p_1_* | = *p_1_* | = *p_1_* | = *p_1_* | 0.94 (0.05) | 0.78 (0.12) | 0.87 (0.088) | 0.72 (0.128) |

|  | *Occupancy (𝜓)* | | | | |  |
| --- | --- | --- | --- | --- | --- | --- |
| Species | *𝜓_1_* | *𝜓_2_* | *𝜓_3_* | *𝜓_4_* | *𝜓_5_* | *Model deviance* |
| *Cuniculus paca* | 0.49 (0.081) | 0.53 (0.077) | 0.44 (0.079) | 0.24 (0.068) | 0.34 (0.066) | 687.65 (22.159) |
| *Dasyprocta punctata* | 0.66 (0.03) | 0.44 (0.062) | 0.34 (0.068) | 0.36 (0.067) | 0.46 (0.069) | 850.76 (20.285) |
| *Dasypus novemcinctus* | 0.62 (0.072) | 0.64 (0.079) | 0.37 (0.078) | 0.36 (0.076) | 0.25 (0.061) | 810.41 (19.483) |
| *Eira barbara* | 0.09 (0.064) | 0.09 (0.045) | 0.09 (0.048) | 0.09 (0.051) | 0.09 (0.053) | 87.58 (12.996) |
| *Leopardus pardalis* | 0.67 (0.082) | 0.53 (0.147) | 0.39 (0.154) | 0.22 (0.128) | 0.45 (0.158) | 272.96 (13.395) |
| *Leopardus wiedii* | 0.14 (0.206) | 0.19 (0.244) | 0.2 (0.215) | 0.32 (0.24) | 0.44 (0.228) | 66.04 (8.048) |
| *Mazama temama* | 0.29 (0.075) | 0.31 (0.061) | 0.31 (0.112) | 0.45 (0.08) | 0.36 (0.085) | 519.01 (22.851) |
| *Nasua Narica* | 0.22 (0.095) | 0.24 (0.086) | 0.23 (0.082) | 0.13 (0.065) | 0.11 (0.056) | 182.37 (16.042) |
| *Panthera Onca* | 0.06 (0.134) | 0.13 (0.204) | 0.25 (0.252) | 0.23 (0.236) | 0.31 (0.242) | 24.07 (5.424) |
| *Pecari tajacu* | 0.49 (0.067) | 0.53 (0.067) | 0.44 (0.07) | 0.45 (0.072) | 0.52 (0.066) | 1013.64 (21.476) |
| *Puma concolor* | 0.2 (0.125) | 0.18 (0.083) | 0.35 (0.239) | 0.29 (0.232) | 0.31 (0.231) | 114.35 (10.522) |
| *Puma yaguaroundi* | 0.2 (0.118) | 0.18 (0.091) | 0.33 (0.233) | 0.29 (0.233) | 0.32 (0.233) | 113.98 (10.896) |
| *Tapirus bardii* | 0.49 (0.042) | 0.61 (0.054) | 0.5 (0.084) | 0.54 (0.072) | 0.43 (0.081) | 726.32 (18.668) |

**Text S1. JAGS/WinBUGS code for model ψ (.)γ(year)ɸ(year)p(.) listed in Table 1.**

# Model 𝜓(.)γ(year)ɸ(year)p(.)

model {

# Specify priors

psi1 ~ dunif(0, 1) #first year occupancy

for(i in 1:(nyear-1)) {

phi[i]~ dunif(0, 1) # apparent survival

gamma[i] ~ dunif(0, 1) # colonization

}

p ~ dunif(0, 1) # detection probability

# Ecological submodel: Define state conditional on parameters

for (i in 1:nsite){

z[i,1] ~ dbern(psi1)

for (k in 2:nyear){

muZ[i,k]<- z[i,k-1]*phi[k-1] + (1-z[i,k-1])*gamma[k-1] #z expected in subsequent years

z[i,k] ~ dbern(muZ[i,k])

}

}

# Observation model

for (i in 1:nsite){

for (j in 1:nrep){

for (k in 1:nyear){

muy[i,j,k] <- z[i,k]*p

y[i,j,k] ~ dbern(muy[i,j,k])

y.new[i,j,k] ~ dbern(muy[i,j,k]) # replicate data set for PPC

}

}

}

#Calculations for a PPC of GOF based on detection frequency per year

for (i in 1:nsite){

for(k in 1:nyear){

sum.y[i,k]<-sum(y[i,,k]) # Observed actual data

eval[i,k]<-max(0.01,sum(muy[i,,k])) # Expected data with a floor of 0.01

E[i,k]<-pow((sum.y[i,k] - eval[i,k]),2)/(eval[i,k]+0.01) #Chi-sq discrepancy, actual data set

sum.y.new[i,k] <- max(0.01,sum(y.new[i,,k]))

E.new[i,k] <- pow((sum.y.new[i,k] - eval[i,k]),2)/(eval[i,k]+0.01) # Chi sq disc, perfect data set

}

}

# Derived parameters: sum of chi-sq, population occupancy, growth rate

fit <- sum(E[,])

fit.new <- sum(E.new[,])

psi[1] <- psi1

for (k in 2:nyear){

psi[k] <- psi[k-1]*phi[k-1] + (1-psi[k-1])*gamma[k-1]

lambda[k-1]<-psi[k]/psi[k-1]

}

}

**Text S2. JAGS/WinBUGS code for models ψ (Ele+Can)γ(year)ɸ(year)p(.) and ψ (Ele+Edg)γ(year)ɸ(year)p(.) listed in Table 1.**

# 𝜓(c1+c2)γ(year)ɸ(year)p(.) Model with two additive covariates (no interaction) in first year occupancy

model { # Priors for covariate coefficients and sate/detection parameters

beta0 ~ dnorm(0,0.1)

beta1 ~ dnorm(0,0.1)

beta2 ~ dnorm(0,0.1)

p ~ dunif(0, 1) # detection probability

for(t in 1:(nyear-1)){

phi[t]~ dunif(0, 1) # apparent survival time t

gamma[t] ~ dunif(0, 1) # colonization time t

}

# Ecological submodel: Define state conditional on parameters

for (i in 1:nsite){

logit(psi1[i])<-beta0 + beta1*c1[i] + beta2*c2[i] #First year occupancy depends on covariates

z[i,1] ~ dbern(psi1[i])

for (t in 2:nyear){

muZ[i,t]<- z[i,t-1]*phi[t-1] + (1-z[i,t-1])*gamma[t-1] #Occupancy in subsequent years

z[i,t] ~ dbern(muZ[i,t])

}

}

# Observation model

for (i in 1:nsite){

for (j in 1:nrep){

for (t in 1:nyear){

muy[i,j,t] <- z[i,t]*p

y[i,j,t] ~ dbern(muy[i,j,t])

y.new[i,j,t] ~ dbern(muy[i,j,t]) # new simulated realization under perfect data

}

}

}

#Calculations for a PPC of GOF based on detection frequency per year

for (i in 1:nsite){

for(t in 1:nyear){

sum.y[i,t]<-sum(y[i,,t]) # Observed actual data

eval[i,t]<-max(0.01,sum(muy[i,,t])) # Expected data with a floor of 0.01

E[i,t]<-pow((sum.y[i,t] - eval[i,t]),2)/(eval[i,t]+0.01) #Chi-sq discrepancy, actual data set

sum.y.new[i,t] <- max(0.01,sum(y.new[i,,t]))

E.new[i,t] <- pow((sum.y.new[i,t] - eval[i,t]),2)/(eval[i,t]+0.01) # Chi sq disc, perfect data set

}

}

# Derived parameters: sum of chi-sq, population occupancy, growth rate

fit <- sum(E[,])

fit.new <- sum(E.new[,])

psi[1] <- sum(z[,1])/nsite1

for (t in 2:nyear){

psi[t] <- psi[t-1]*phi[t-1] + (1-psi[t-1])*gamma[t-1]

lambda[t-1]<-psi[t]/psi[t-1]

}

}

**Text S3. JAGS/WinBUGS code for model ψ (.)γ(year)ɸ(year)p(year) listed in Table 1.**

model {

# 𝜓(.)γ(year)ɸ(year)p(year) Model with year-specific survival, colonization and detection

# Specify priors

psi1 ~ dunif(0, 1) #first year occupancy

for(i in 1:(nyear-1)) {

phi[i]~ dunif(0, 1) # apparent survival

gamma[i] ~ dunif(0, 1) # colonization

p[i] ~ dunif(0, 1) # detection

}

p[nyear] ~ dunif(0, 1) # detection last year

# Ecological submodel: Define state conditional on parameters

for (i in 1:nsite){

z[i,1] ~ dbern(psi1)

for (k in 2:nyear){

muZ[i,k]<- z[i,k-1]*phi[k-1] + (1-z[i,k-1])*gamma[k-1]

z[i,k] ~ dbern(muZ[i,k])

}

}

# Observation model

for (i in 1:nsite){

for (j in 1:nrep){

for (k in 1:nyear){

muy[i,j,k] <- z[i,k]*p[k]

y[i,j,k] ~ dbern(muy[i,j,k])

y.new[i,j,k] ~ dbern(muy[i,j,k]) # replicate data set for PPC

}

}

}

#Calculations for a PPC of GOF based on detection frequency per year

for (i in 1:nsite){

for(k in 1:nyear){

sum.y[i,k]<-sum(y[i,,k]) # Observed actual data

eval[i,k]<-max(0.01,sum(muy[i,,k])) # Expected data with a floor of 0.01

E[i,k]<-pow((sum.y[i,k] - eval[i,k]),2)/(eval[i,k]+0.01) #Chi-sq discrepancy, actual data set

sum.y.new[i,k] <- max(0.01,sum(y.new[i,,k]))

E.new[i,k] <- pow((sum.y.new[i,k] - eval[i,k]),2)/(eval[i,k]+0.01) # Chi sq disc, perfect data set

}

}

# Derived parameters: sum of chi-sq, population occupancy, growth rate

fit <- sum(E[,])

fit.new <- sum(E.new[,])

psi[1] <- psi1

for (k in 2:nyear){

psi[k] <- psi[k-1]*phi[k-1] + (1-psi[k-1])*gamma[k-1]

lambda[k-1]<-psi[k]/psi[k-1]

}

}

**Text S4. JAGS/WinBUGS code for model 𝜓ψ(.)γ(.)ɸ(.)p(.) listed in Table 1.**

# model 𝜓(.)γ(.)ɸ(.)p(.) with all parameters constant

model {

# Specify priors

psi1 ~ dunif(0, 1) #first year occupancy

phi~ dunif(0, 1) # apparent survival

gamma ~ dunif(0, 1) # colonization

p ~ dunif(0, 1) # detection

# Ecological submodel: Define state conditional on parameters

for (i in 1:nsite){

z[i,1] ~ dbern(psi1)

for (k in 2:nyear){

muZ[i,k]<- z[i,k-1]*phi + (1-z[i,k-1])*gamma

z[i,k] ~ dbern(muZ[i,k])

}

}

# Observation model

for (i in 1:nsite){

for (j in 1:nrep){

for (k in 1:nyear){

muy[i,j,k] <- z[i,k]*p

y[i,j,k] ~ dbern(muy[i,j,k])

y.new[i,j,k] ~ dbern(muy[i,j,k]) # replicate data set for PPC

}

}

}

#Calculations for a PPC of GOF based on detection frequency per year

for (i in 1:nsite){

for(k in 1:nyear){

sum.y[i,k]<-sum(y[i,,k]) # Observed actual data

eval[i,k]<-max(0.01,sum(muy[i,,k])) # Expected data with a floor of 0.01

E[i,k]<-pow((sum.y[i,k] - eval[i,k]),2)/(eval[i,k]) #Chi-sq discrepancy, actual data set

sum.y.new[i,k] <- max(0.01,sum(y.new[i,,k]))

E.new[i,k] <- pow((sum.y.new[i,k] - eval[i,k]),2)/(eval[i,k]) # Chi sq disc, perfect data set

}

}

# Derived parameters: sum of chi-sq, population occupancy, growth rate

fit <- sum(E[,])

fit.new <- sum(E.new[,])

psi[1] <- psi1

for (k in 2:nyear){

psi[k] <- psi[k-1]*phi + (1-psi[k-1])*gamma

lambda[k-1]<-psi[k]/psi[k-1]

}

}

**Text S5. JAGS/WinBUGS code for model 𝜓ψ(c1)γ(year)ɸ(year)p(.) listed in Table 1.**

# model 𝜓(c1)γ(year)ɸ(year)p(.) – one covariate on first year occupancy

model {

#priors for covariate coefficients

beta0 ~ dnorm(0,0.1)

beta1 ~ dnorm(0,0.1)

#priors for model parameters

p ~ dunif(0, 1) # detection probability

for(t in 1:(nyear-1)){

phi[t]~ dunif(0, 1) # apparent survival time t

gamma[t] ~ dunif(0, 1) # colonization time t

}

# Ecological submodel: Define state conditional on parameters

for (i in 1:nsite){

logit(psi1[i])<-beta0 + beta1*c1[i]

z[i,1] ~ dbern(psi1[i])

for (t in 2:nyear){

muZ[i,t]<- z[i,t-1]*phi[t-1] + (1-z[i,t-1])*gamma[t-1]

z[i,t] ~ dbern(muZ[i,t])

}

}

# Observation model

for (i in 1:nsite){

for (j in 1:nrep){

for (t in 1:nyear){

muy[i,j,t] <- z[i,t]*p

y[i,j,t] ~ dbern(muy[i,j,t])

y.new[i,j,t] ~ dbern(muy[i,j,t]) # new simulated realization under perfect data

}

}

}

#Calculations for a PPC of GOF based on detection frequency per year

for (i in 1:nsite){

for(t in 1:nyear){

sum.y[i,t]<-sum(y[i,,t]) # Observed actual data

eval[i,t]<-max(0.01,sum(muy[i,,t])) # Expected data with a floor of 0.01

E[i,t]<-pow((sum.y[i,t] - eval[i,t]),2)/(eval[i,t]+0.01) #Chi-sq discrepancy, actual data set

sum.y.new[i,t] <- max(0.01,sum(y.new[i,,t]))

E.new[i,t] <- pow((sum.y.new[i,t] - eval[i,t]),2)/(eval[i,t]+0.01) # Chi sq disc, perfect data set

}

}

# Derived parameters: sum of chi-sq, population occupancy, growth rate

fit <- sum(E[,])

fit.new <- sum(E.new[,])

psi[1] <- sum(z[,1])/nsite1

for (t in 2:nyear){

psi[t] <- psi[t-1]*phi[t-1] + (1-psi[t-1])*gamma[t-1]

lambda[t-1]<-psi[t]/psi[t-1]

}

}

**Table S4. Parameter and effects for species richness model**. The final model had the following structure: ψ(.)γ(.)ɸ(.)p(BS+guild+year). BS = body size; guild = herbivore, carnivore, omnivore or insectivore; year = time dependent covariate. Covariates in p() were fit using a means parameterization.

| Parameter (symbol) | Covariates/levels | Effect estimate | Standard error |
| --- | --- | --- | --- |
| Occupancy year 1 (Ψ) | – | 0.402 | 0.0778 |
| Colonization (γ) | – | 0.151 | 0.0393 |
| Survival (ɸ) | – | 0.817 | 0.0555 |
| Detection (p) | Body size | 0.506 | 0.0066 |
|  | Guild_carnivore | 0.111 | 0.0155 |
|  | Guild_herbivore | 0.333 | 0.0221 |
|  | Guild_insectivore | 0.275 | 0.0266 |
|  | Guild_omnivore | 0.084 | 0.0110 |
|  | Year_2009 | 0.493 | 0.0313 |
|  | Year_2010 | 0.465 | 0.0358 |
|  | Year_2011 | 0.413 | 0.0373 |
|  | Year_2012 | 0.356 | 0.0294 |
